# Mitogenomic phylogeny of Truncatelloidea with description of *Aenigmula sinensis* Tang, Han & Kong, sp. nov. (Mollusca, Gastropoda, Littorinimorpha, Truncatelloidea)

**DOI:** 10.3897/zookeys.1279.183841

**Published:** 2026-05-19

**Authors:** Jiaze Tang, Jihang Gong, Xiao Han, Yan Fan, Xinni Zhang, Lingfeng Kong

**Affiliations:** 1 Key Laboratory of Mariculture, Ministry of Education, Ocean University of China, Qingdao 266003, China Key Laboratory of Mariculture, Ministry of Education, Ocean University of China Qingdao China; 2 Laboratory for Marine Fisheries Science and Food Production Processes, Qingdao Marine Science and Technology Center, Qingdao, Shandong 266237, China Laboratory for Marine Fisheries Science and Food Production Processes, Qingdao Marine Science and Technology Center Qingdao China

**Keywords:** *
Aenigmula
*, China, morphology, new species, phylogenetics, taxonomy

## Abstract

Truncatelloidea is the richest and most diverse group within Mollusca. Both morphological and molecular studies have sought to resolve its phylogenetic framework; however, the phylogenetic relationships among some lineages remain controversial. To explore the phylogenetic relationships within Truncatelloidea, this study presents a mitochondrial phylogenomic framework, reconstructed from a dataset integrating 15 newly sequenced mitochondrial genomes with existing data from NCBI and encompassing 20 genera from 11 families. Both maximum likelihood and Bayesian inference analyses supported that the superfamily was resolved into two major clades (I and II). Within Clade I, Assimineidae and Stenothyridae form a sister group, while Baicaliidae and Bithyniidae are distantly related to the remaining families. Within Clade II, *Aenigmula* and Clenchiellidae form a sister group, with Iravadiidae occupying the most basal position. Furthermore, the monophyly of all other families and genera is strongly supported except for *Assiminea* within Assimineidae. Additionally, based on morphological observations, phylogenetic analyses of COI, 16S rRNA, and 28S rRNA gene fragments, species delimitation methods (ABGD, ASAP, bPTP), and genetic distance calculations of the collected Truncatelloidea specimens, a new species, *Aenigmula sinensis* sp. nov., was identified and described, while an undetermined species, *Aenigmula* sp., was recorded. The findings strongly support the recognition of *Aenigmula* as an independent lineage, offering more comprehensive morphological character descriptions and molecular evidence for this genus.

## Introduction

Truncatelloidea is one of the largest and most diverse superfamilies within Mollusca, comprising 37 families (two of which are extinct) and 715 genera. Its members are primarily micro- or small-sized mollusks, with body sizes ranging from 0.5 mm (e.g., Spirostyliferinidae, Moitessieriidae) to 15 mm (e.g., Bithyniidae, Hydrobiidae), and they are widely distributed across marine, freshwater, and terrestrial environments (Kuroda 1962; Lima et al. 2016; Osorno-Arango and Cantera-Kintz 2021).

In early studies, the boundaries of taxonomic units within Truncatelloidea were poorly defined due to homogeneity in shell morphology and anatomical features (Davis 1979; Hershler and Ponder 1998). With advances in molecular systematics, Criscione and Ponder (2013) formally established the monophyly of Truncatelloidea based on molecular evidence. Subsequently, significant progress has been made at the genus and species levels, particularly within families such as Bithyniidae (Ting and Siong 2024), Cochliopidae (Collado et al. 2016; Diaz et al. 2020), Hydrobiidae (Delicado et al. 2016; Diana et al. 2021; Jaszczyńska et al. 2023), Moitessieriidae (Richling et al. 2017; Hofman et al. 2018), and Pomatiopsidae (Salvador et al. 2022). However, systematic studies at the family level and above remain notably limited. Phylogenetic analyses primarily rely on short genetic segments, and consistent results for the phylogenetic positions of many families within Truncatelloidea remain elusive. This is particularly problematic for families including Anabathridae, Caecidae, Iravadiidae, and Tornidae (Criscione and Ponder 2013; Golding 2014b; Layton et al. 2019; Goto et al. 2021; Zhang et al. 2024). Mitochondrial genomes, owing to their characteristics of providing additional gene loci and containing rich molecular sequence and gene structural information, have been demonstrated to effectively enhance the resolution and statistical confidence of phylogenetic trees, becoming a powerful tool for resolving phylogenetic relationships among diverse metazoans (Mueller 2006; Li et al. 2012; Osigus et al. 2013; Mabuchi et al. 2014; Varney et al. 2021; Liu et al. 2022; Xu et al. 2024; Li et al. 2025). Consequently, constructing datasets that incorporate mitochondrial genomes from representative taxa is a promising approach for clarifying the phylogenetic relationships within this challenging group.

The widespread application of molecular methods has not only advanced phylogenetic studies but also significantly accelerated the discovery of new taxa (Criscione and Ponder 2013; Golding 2014a, 2014b; Ponder et al. 2014; Takano and Kano 2014; Layton et al. 2019; Goto et al. 2021; Zhang et al. 2024). *Aenigmula criscionei* Golding, 2014, from Australia’s Northern Territory, was proposed by Golding (2014b), one of the earliest researchers to apply molecular methods to Truncatelloidea. The genus is characterized by typical truncatelloidean features: small, ovoid to conical shells lacking sculpture, a teardrop-shaped aperture, a dome-shaped protoconch, a paucispiral operculum, and curved, elongated lateral teeth. Despite molecular phylogenetic analyses suggesting a close relationship between *Aenigmula* and families within Truncatelloidea such as Caecidae, Clenchiellidae, and Calopiidae, its phylogenetic position, genetic distance from these families, and morphological characteristics differ significantly. Therefore, Golding (2014b) concluded that a new genus was warranted for *A. criscionei*, without assigning it to any existing family. Subsequently, *Aenigmula* has been used as an outgroup in phylogenetic analyses of families such as Caecidae and Clenchiellidae (Ponder et al. 2014; Egger et al. 2020). To date, *A. criscionei* remains the only described species in the genus, highlighting the need for increased sampling to accurately determine its taxonomic status and phylogenetic affinities.

In this study, we first aimed to construct a phylogenetic framework for Truncatelloidea using mitochondrial genomes to resolve higher-level relationships. We collected samples of micromolluscs belonging to Truncatelloidea along the Chinese coast, obtaining specimens from ten genera across seven families, and successfully sequenced 15 new mitochondrial genomes. Subsequently, among these collections, an undescribed species was identified as belonging to the genus *Aenigmula*. Based on morphological observations and phylogenetic trees constructed from short segments of COI, 16S rRNA, and 28S rRNA genes, this new species is formally described as *Aenigmula sinensis* sp. nov. Another single specimen of this genus was identified as *Aenigmula* sp. The mitochondrial genome data also enabled us to determine the phylogenetic position of *Aenigmula* within the superfamily.

## Materials and methods

### Sampling and preservation

**Figure 1.**
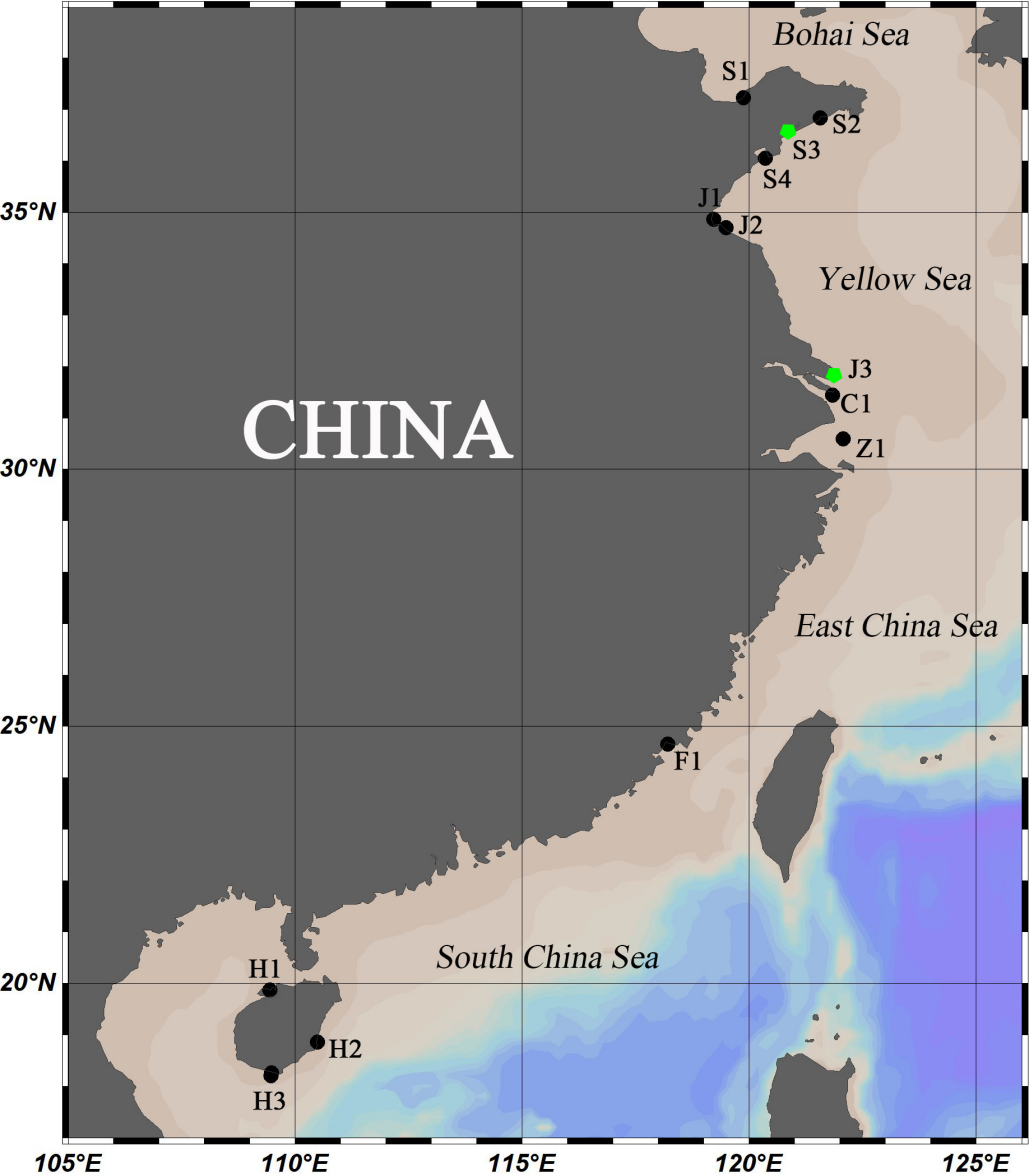
Map of sampling sites. Green pentagrams mark the type locality of *A. sinensis* sp. nov. (S3) and the collection site for *A.* sp. (J3).

Samples were collected from coastal regions of China (Fig. 1) and preserved using different methods depending on the experimental purpose (Geiger et al. 2007). For molecular research, specimens were immersed in 95% ethanol and stored at – 30 °C. For microscopic observation, specimens were briefly immersed in a 5%–10% formalin solution (prepared with seawater, pH 7.0), followed by preservation in 70% ethanol at – 80 °C. The type specimens of the new species were deposited at the Laboratory of Shellfish Genetics and Breeding (**LSGB**) of Ocean University of China.

### Morphological observation and identification

Standard shell views were captured using a Nikon SMZ 800N stereomicroscope with parallel incident illumination. Image stacks were processed with HELICON FOCUS 8.0.2 software for focus stacking to generate composite images with an extended depth of field. Radulae were treated with 10% KOH and proteinase K at 30–50 °C to remove soft tissues, and washed with ultrapure water before scanning electron microscopy (SEM). Opercula were cleaned with 15% diluted bleach and soaked in 70% ethanol before SEM observation. All shells, radulae, and opercula were coated with gold prior to examination.

### DNA extraction and sequencing

Genomic DNA was extracted using the TIANamp Marine Animal DNA Kit (TIANGEN). DNA quality was assessed via 1% agarose gel electrophoresis, and the DNA was stored at – 20 °C for subsequent use.

DNA extracted from four individuals of the new species was amplified by polymerase chain reaction (PCR) for the COI, 16S rRNA, and 28S rRNA. The amplifications were conducted in a total volume of 25 μL, including 1 μL of DNA template, 22 μL of Gold Mix (Tsingke), and 1 μL each of forward and reverse primers. The primers and PCR conditions used in the present study are listed in Table 1. PCR products were analyzed by 1.5% agarose gel electrophoresis before being sent to BGI Genomics for purification and bidirectional sequencing. To obtain the mitochondrial genomes of the collected samples, the extracted DNA was sent to Novogene for library construction and sequencing, with an average insert size of ~ 300 bp. Sequencing was performed on the Illumina NovaSeq 6000 platform using a PE150 strategy, generating ~ 8 Gb of raw data per library.

**Table 1.** Primers and PCR conditions used in the present study.

Gene	Primer name	Nucleotide sequence(5'-3')	References
COI	LCO1490	GGTCAACAAATCATAAAGATATTGG	Folmer et al. (1994)
	HCO2198	TAAACTTCAGGGTGACCAAAAAATCA	
16S	16SARis	TGCCTGTTTAGCAAAAACAT	Criscione and Ponder (2013)
	16SBRis	CCGGTCTGAACTCAGATCATGT	
28S	28SDK	GATCGGACGAGATTACCCGCTGAA	Strong et al. (2011) / Williams et al. (2003)
	LSU1600R	AGCGCCATCCATTTTCAGG	
Fragment	PCR conditions		
COI	94 °C(3') [x1]; 94 °C(45"), 48 °C(45"), 72 °C(1') [x37]; 72 °C(7') [x1]		
16S	94 °C(5'), 52 °C(30"), 72 °C(1') [x1]; 94 °C(30"), 52 °C(30"), 72 °C(1') [x40]; 94 °C (30"), 52 °C(30"),72 °C(7') [x1]		
28S	94 °C(3') [x1]; 94 °C(45"), 58 °C(45"), 72 °C(1') [x37]; 72 °C(7') [x1]		

### Species delimitation

Three species delimitation methods were employed for the newly collected specimens. The first was Automatic Barcode Gap Discovery (ABGD) (Puillandre et al. 2012). The alignment of the fast-evolving COI gene was submitted to the ABGD web platform for analysis under the Jukes-Cantor (JC69) model and default settings. The second was Assemble Species by Automatic Partitioning (ASAP) (Puillandre et al. 2021). COI sequences were input into the ASAP platform, with genetic distances calculated under the Kimura 2-parameter (K2P) model, and the partition with the lowest ASAP score was selected as the result. The third was Bayesian Poisson Tree Process (bPTP) (Zhang et al. 2013). The maximum likelihood (ML) tree generated by RAxML was uploaded to the bPTP web server (https://species.h-its.org/ptp) for analysis.

### Mitochondrial genome assembly and annotation

The raw data were processed using Trimmomatic 0.36 (Bolger et al. 2014) and subsequently assembled with either SPAdes v. 3.15.4 (Bankevich et al. 2012) or GetOrganelle v. 1.7.7.0 (Jin et al. 2020). The protein-coding genes (PCGs) were annotated using the Open Reading Frame Finder (https://www.ncbi.nlm.nih.gov/orffinder/) and the MITOS web server (Bernt et al. 2013) with default settings and the invertebrate mitochondrial genetic code. Annotations were then manually verified and corrected against related mitogenomes from GenBank using Artemis (Rutherford et al. 2000) to refine gene boundaries. The tRNA genes were identified using ARWEN (Laslett and Canbäck 2008) and tRNAscan-SE (Lowe and Eddy 1997). The rRNA genes were confirmed by comparison with published truncatelloidean mitochondrial genomes, with their boundaries inferred from adjacent genes (Boore et al. 2005). The complete mitochondrial genome sequences have been deposited in GenBank.

### Dataset construction

For this study, we obtained four sets of COI, 16S rRNA, and 28S rRNA genes from *Aenigmula sinensis* sp. nov., along with a COI sequence from *Aenigmula* sp. Based on previous research (Golding 2014b; Ponder et al. 2014; Egger et al. 2020), short fragment sequences from four closely related families obtained from GenBank (Suppl. material 1: table SS1) were incorporated to conduct the phylogenetic analysis and delimitation of the new species. Additionally, a dataset comprising 15 newly sequenced mitochondrial genomes (including one outgroup) and published Truncatelloidea mitogenomes from GenBank (Suppl. material 1: table SS2) was assembled to reconstruct the phylogeny of Truncatelloidea. The phylogenetic relationship was inferred from the concatenated amino acid sequences of 13 mitochondrial protein-coding genes, using one species from Rissooidea as the outgroup (Criscione and Ponder 2013; Golding 2014b).

Both datasets were aligned using MAFFT (Katoh and Standley 2013). The aligned sequences were trimmed with Gblocks (Talavera and Castresana 2007) to remove ambiguous sites, using default parameters. The resulting datasets were then used to construct corresponding concatenated gene datasets for phylogenetic analysis using FASconCAT (Kück and Meusemann 2010). Genetic distances among species within the genus *Aenigmula* were analyzed using MEGA11 (Tamura et al. 2021).

### Phylogenetic analysis

PartitionFinder 2 (Lanfear et al. 2017) was first employed to determine the optimal partitioning schemes and substitution models for the datasets based on the Bayesian information criterion (BIC). Subsequently, maximum likelihood (ML, Felsenstein 1981) and Bayesian inference (BI, Huelsenbeck and Ronquist 2001) were used to reconstruct phylogenetic relationships. ML analyses were conducted using IQ-TREE v. 1.6.8 (Nguyen et al. 2015) with 1000 bootstrap replicates. BI analyses were performed using MrBayes v. 3.2.7a (Ronquist et al. 2012) with four simultaneous Markov chain Monte Carlo (MCMC) chains run for 10 million generations, sampling every 1000 generations and discarding the first 25% of samples as burn-in. Parameter convergence was achieved within 10 million generations, with a standard deviation of split frequencies below 0.01. All parameters were checked using Tracer v. 1.7 (Rambaut et al. 2018), revealing effective sample size (ESS) values greater than 200. The final phylogenetic trees were visualized and edited in FigTree v. 1.4.4 (Rambaut 2014).

## Results

### Phylogenetic relationships within Truncatelloidea

**Figure 2.**
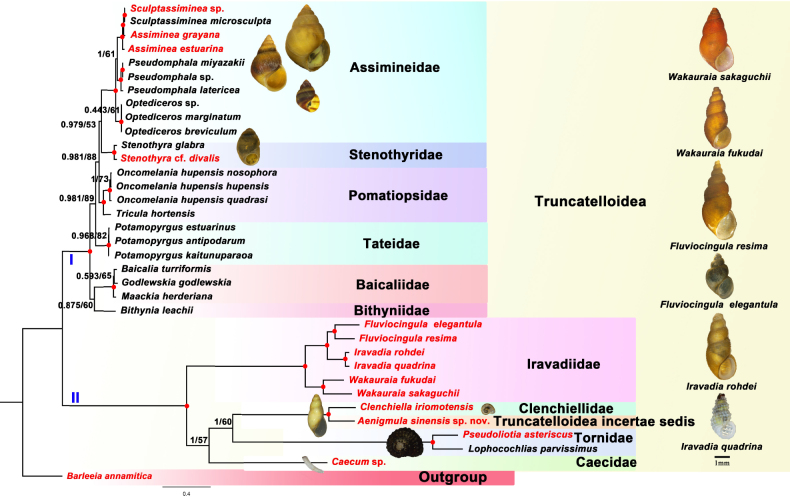
Phylogenetic tree inferred by Bayesian analysis (BI) and maximum likelihood (ML) based on concatenated amino acids of 13 mitochondrial protein-coding genes. The BI phylograms are shown. Numbers at nodes are statistical support values for BI (posterior probabilities)/ML (bootstrap proportions in percentage). Solid red circles represent nodes with posterior probabilities ≥ 0.95 and bootstrap proportions ≥ 90. The newly sequenced mitochondrial (mt) genomes are indicated in red.

A total of 34 taxa, comprising 20 genera from 11 families within Truncatelloidea, were selected for phylogenetic analyses, producing a phylogenetic tree that represents the most extensive mitogenomic dataset of Truncatelloidea to date. The best partition scheme for the dataset (containing 3699 sites) was determined based on subunit combinations, with each gene treated as a fit partition except for *atp6-atp8* and *nad4-nad4L*. The optimal substitution models were as follows: LG+G+F for *atp6-atp8*, MTART+I+G for *cob*, MTART+I+G+F for *cox1-2-3*, and LG+I+G+F for *nad1-2-3-4-4L-5-6*. Phylogenetic analyses using both ML and BI methods yielded almost identical topologies. In the BI tree, nearly all nodes were strongly supported, whereas several nodes in the ML tree exhibited moderate to low support (Fig. 2).

Phylogenetic relationships of Truncatelloidea are consistently resolved into two clades (I and II) with the highest support (BI posterior probability PP = 1; ML bootstrap support BS = 100). Clade I includes Assimineidae, Baicaliidae, Bithyniidae, Pomatiopsidae, Stenothyridae, and Tateidae, while Clade II encompasses Tornidae, Clenchiellidae, Caecidae, and Iravadiidae. Within Clade I, two subclades are recovered with strong support (PP = 1; BS = 100%). One contains Baicaliidae and Bithyniidae as sister families (PP = 0.875; BS = 60%). In the other subclade, Assimineidae and Stenothyridae form a sister group (PP = 0.979; BS = 53%) that is sister to Pomatiopsidae (PP = 0.981; BS = 88%). Subsequently, these three families group with Tateidae (PP = 0.981; BS = 89%). Within Clade II, the new species *A. sinensis* sp. nov. forms a sister taxon to Clenchiellidae (PP = 1; BS = 100%); this clade is sister to Tornidae (PP = 1; BS = 60%), then clusters with Caecidae (PP = 1; BS = 57%), and finally with Iravadiidae (PP = 1; BS = 100%). The phylogenetic relationships among all genera and species within Iravadiidae received the highest support (PP = 1; BS = 100%). Except for *Assiminea* within Assimineidae, the monophyly of all other families and genera is strongly supported.

### New species

**Figure 3.**
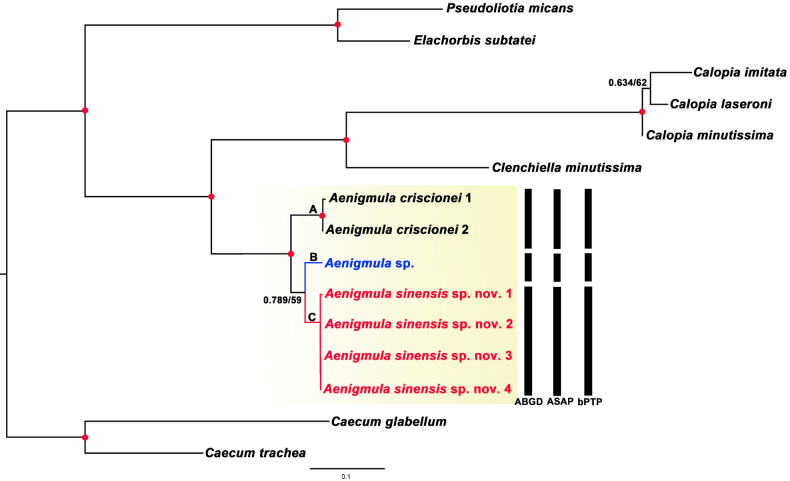
Phylogenetic tree inferred by Bayesian analysis (BI) and maximum likelihood (ML) based on concatenated dataset of COI, 16S and 28S genes. Numbers at nodes are statistical support values for BI (posterior probabilities)/ML (bootstrap proportions in percentage). Solid red circles represent nodes with posterior probabilities ≥ 0.95 and bootstrap proportions ≥ 90. the yellow shaded area highlights the clade of *Aenigmula*. The bars on the right side of the phylogram are three species delimitation methods (in order ABGD, ASAP, and bPTP).

The phylogenetic trees reconstructed from the concatenated sequences of COI, 16S, and 28S rRNA genes using both ML and BI methods yielded almost identical topologies (Fig. 3). The *Aenigmula* clade was recovered with the highest support (PP = 1; BS = 100%) and was clearly divergent from all other families. Within this genus, three species were distinctly separated in the phylogenetic tree. *A.* sp. and *A. sinensis* sp. nov. formed a sister group, though this node received lower support (PP = 0.789; BS = 59%), perhaps due to the limited data available for *A.* sp. (COI only). Subsequently, this clade clustered with *A. criscionei* with strong support (PP = 1; BS = 99%). All three species delimitation methods (ABGD, ASAP, and bPTP) consistently identified *A. criscionei*, *A. sinensis* sp. nov., and *A.* sp. as distinct molecular operational taxonomic units (Fig. 3).

Analysis of interspecific Kimura 2-parameter pairwise distances based on COI sequences (Table 2) revealed that the genetic distance between *A. criscionei* and *A.* sp. was 13.7%, while distances ranged from 12.4% to 12.8% between *A. criscionei* and *A. sinensis* sp. nov., and 8.3% to 8.9% between *A.* sp. and *A. sinensis* sp. nov. These values substantially exceeded the observed average intraspecific distances (0–1.22%), providing further evidence for the distinctness of the three species.

**Table 2.** Kimura 2-parameter pairwise distance among *Aenigmula* species based on COI sequences.

**Species label**	**1**	**2**	**3**	**4**	**5**	**6**
1 *A. criscionei* 1						
2 *A. criscionei* 2	0.01216					
3 *A.* sp.	0.14283	0.13888				
4 *A. sinensis* sp. nov. 1	0.12434	0.12810	0.09351			
5 *A. sinensis* sp. nov. 2	0.12810	0.13189	0.09351	0.00909		
6 *A. sinensis* sp. nov. 3	0.12434	0.12810	0.08623	0.00604	0.00604	
7 *A. sinensis* sp. nov. 4	0.11483	0.11892	0.08644	0.00671	0.00503	0.00334

### Systematics

#### Order Littorinimorpha Golikov & Starobogatov, 1975

**Superfamily Truncatelloidea J. E. Gray, 1840**

##### Genus Aenigmula

Taxon classificationAnimaliaLittorinimorphaTornidae

Golding, 2014

11017FB5-8E60-57FE-B6DB-ECCB2FAD9AC5

###### Diagnosis (Golding, 2014b).

Shell small, glossy, ovate-conic, unsculptured, non-umbilicate; teardrop-shaped aperture lacking external varix. Protoconch dome-shaped. Operculum paucispiral, nucleus eccentric. Radula with curved, elongate lateral margins, single pair of basal denticles present.

###### Type species.

*Aenigmula criscionei* Golding, 2014, by original designation.

###### Type locality.

Rapid Creek, Darwin.

##### Aenigmula sinensis

Taxon classificationAnimaliaLittorinimorphaTornidae

Tang, Han & Kong sp. nov.

68EB9BE4-6E7E-5218-9F49-4040CA60053C

https://zoobank.org/DB669881-543F-418D-82AC-783B5E0ABCC6

Figs 4, 5, 6

###### Type material.

**Figure 4.**
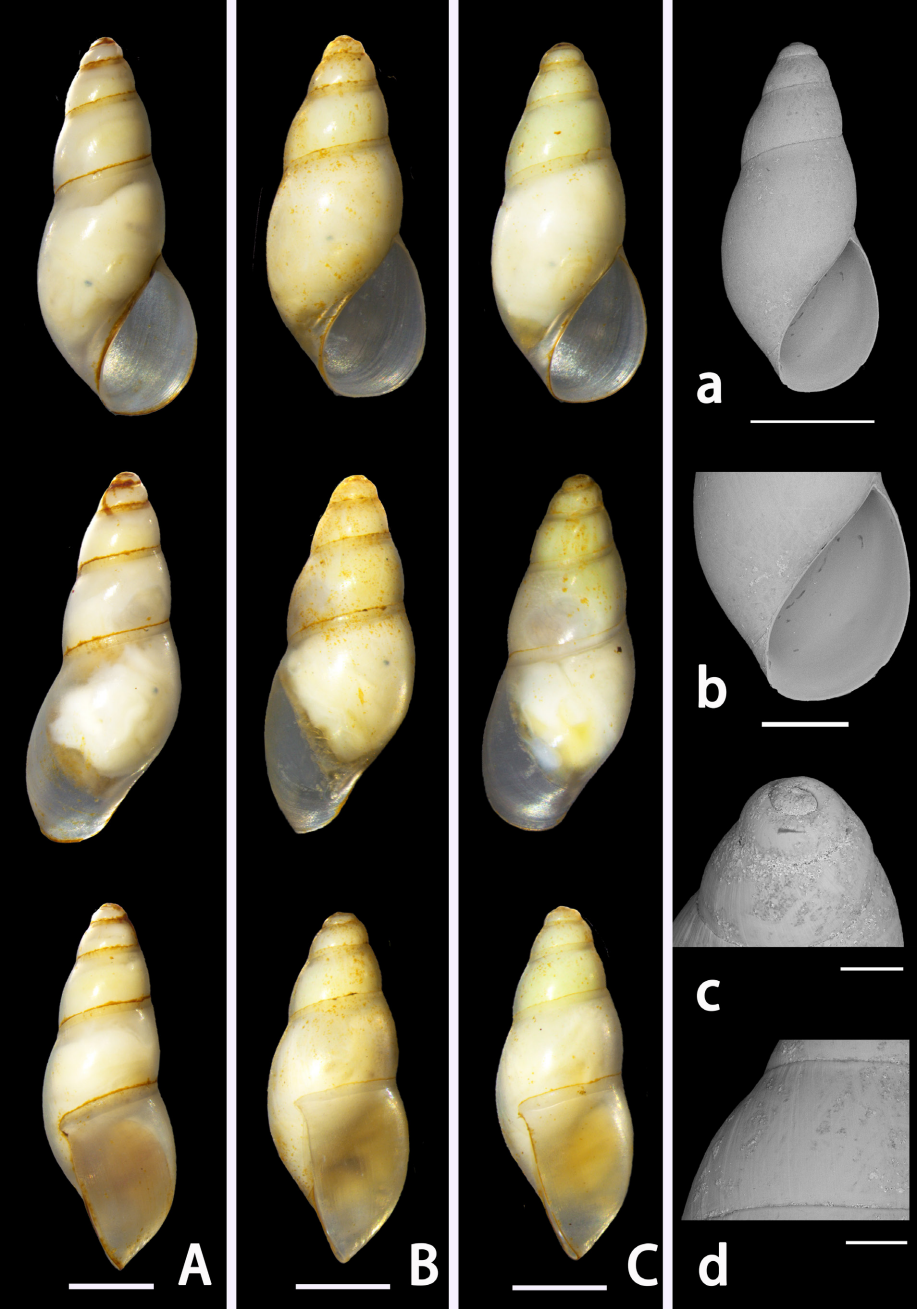
*
Aenigmula sinensis* sp. nov. **A**. Holotype; **B, C**. Paratype; **a–d**. Scanning electron micrographs: **a**. Apertural view of shell; **b**. Aperture region; **c**. Protoconch; **d**. Shell surface sculpture. Scale bars: 1000 μm (**A–C, a**); 500 μm (**b–d**).

***Holotype*** (Fig. 4A). 1 specimen; Qikou Village, Jimo District, Qingdao, Shandong, China; 36°36'12"N, 120°50'20"E; coll. Jiaze Tang; 27 March 2024; accession no. LSGB-mg266200-0901. ***ParIatypes*** (Fig. 4B, C). 2 specimens; same collection data as holotype; LSGB-mg266200-0902, LSGB-mg266200-0903.

###### Measurements.

(Table 3)

**Table 3.** Measurements of *Aenigmula sinensis* sp. nov. (μm). Abbreviations: W, shell width; H, shell height; LA, length of aperture; WA, width of aperture.

	Number	H	W	LA	WA
**Holotype**	LSGB-mg266200-0901	3266	1076	1270	753
**Paratype 1**	LSGB-mg266200-0902	2570	886	1095	588
**Paratype 2**	LSGB-mg266200-0903	2566	976	1117	699

###### Diagnosis.

Shell elongate-conic, pale yellow and translucent; whorls convex, smooth except for growth lines, devoid of other sculpture, imperforate, protoconch dome-shaped. Aperture teardrop-shaped with one straight side, lacking external varix. Radula with main cusp of central tooth markedly prominent, nearly level with the single pair of basal denticles; lateral teeth curved, triangular in profile. Operculum paucispiral, with an eccentric nucleus.

###### Description.

***Shell*** (Fig. 4a–d). Small (< 3.5 mm), elongate-conic, thin, pale yellow, and translucent; spire comprising 5–6 whorls, convex, well-inflated; suture distinct, simple. Protoconch dome-shaped. Teleoconch smooth, sculptured only with fine growth lines, lacking other sculpture. Aperture D-shaped; outer lip thin, slightly expanded; inner lip narrow, without external varix. Imperforate.

**Figure 5.**
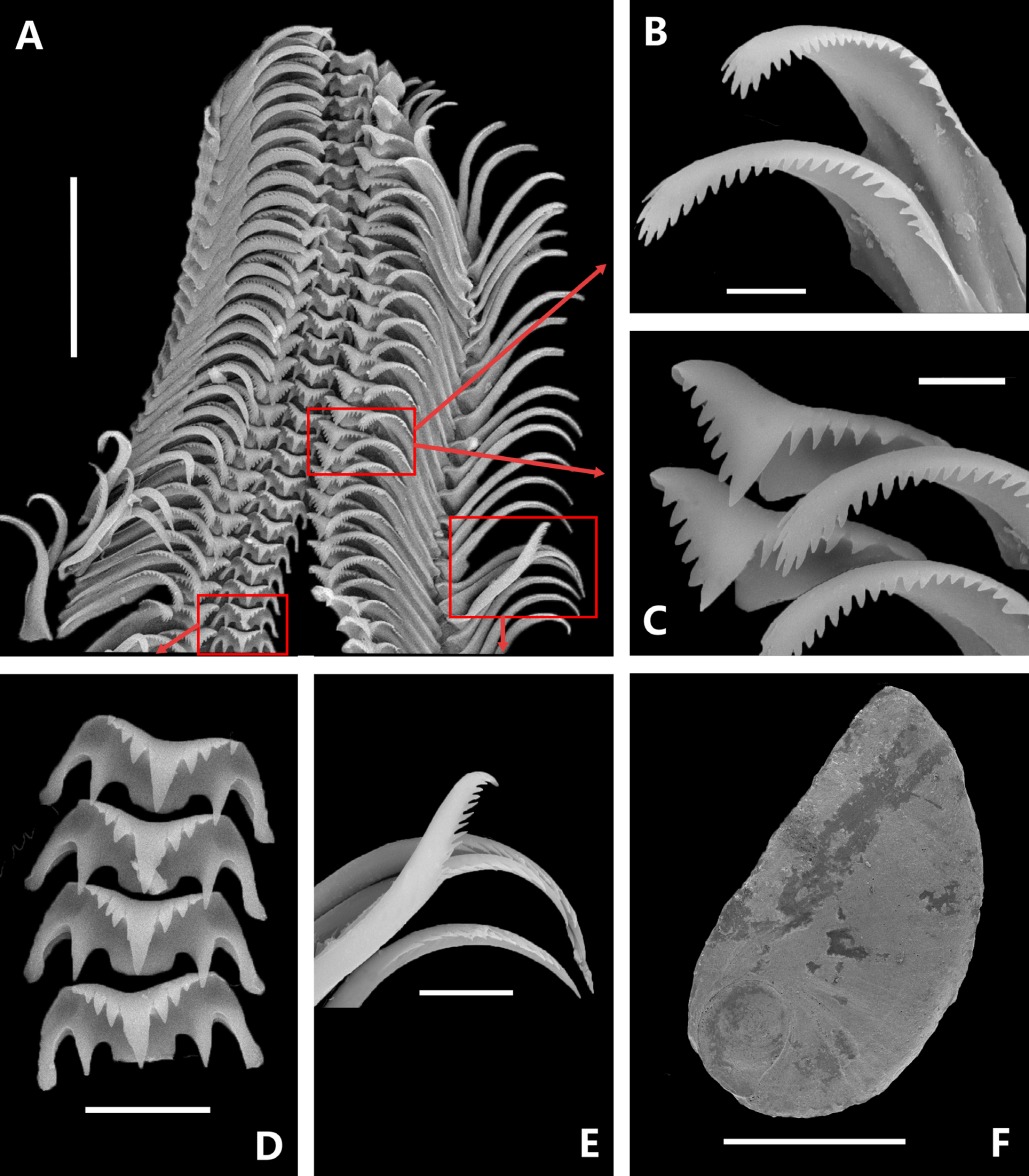
Scanning electron micrographs of the radula and operculum of *Aenigmula sinensis* sp. nov. **A**. Radula; **B**. Inner marginal teeth; **C**. Lateral teeth; **D**. Central tooth; **E**. Outer marginal teeth; **F**. Operculum. Scale bars: 500 μm (**F**); 50 μm (**A**); 10 μm (**D, E**); 5 μm (**B, C**).

***Radula*** (Fig. 5A–E). Central tooth 3+1+3 / 1 1, trapezoidal, bearing a single slender conical main cusp at the apex and three sharp denticles on each side (distinctly shorter than the main cusp and decreasing in length laterally), with one pair of well-developed, elongated conical basal denticles (Fig. 5D). Lateral teeth 5–6+1+5, distinctly asymmetrical; main cusp offset inward; inner denticles comb-like and subequal in size; outer denticles arranged more sparsely and gradually diminishing in size outward (Fig. 5C). Marginal teeth elongated and curved, comprising inner and outer marginal teeth: inner marginal teeth with relatively broad base, bearing 20–22 sharp cusps externally and only 3–5 small denticles internally (Fig. 5B); outer marginal teeth with 10–15 cusps confined to the inner margin (Fig. 5E).

***Operculum*** (Fig. 5F). Operculum D-shaped; paucispiral, with an eccentric nucleus situated near the anterior umbilical margin; the surface exhibits only faint spiral growth lines near the nucleus.

**Figure 6.**
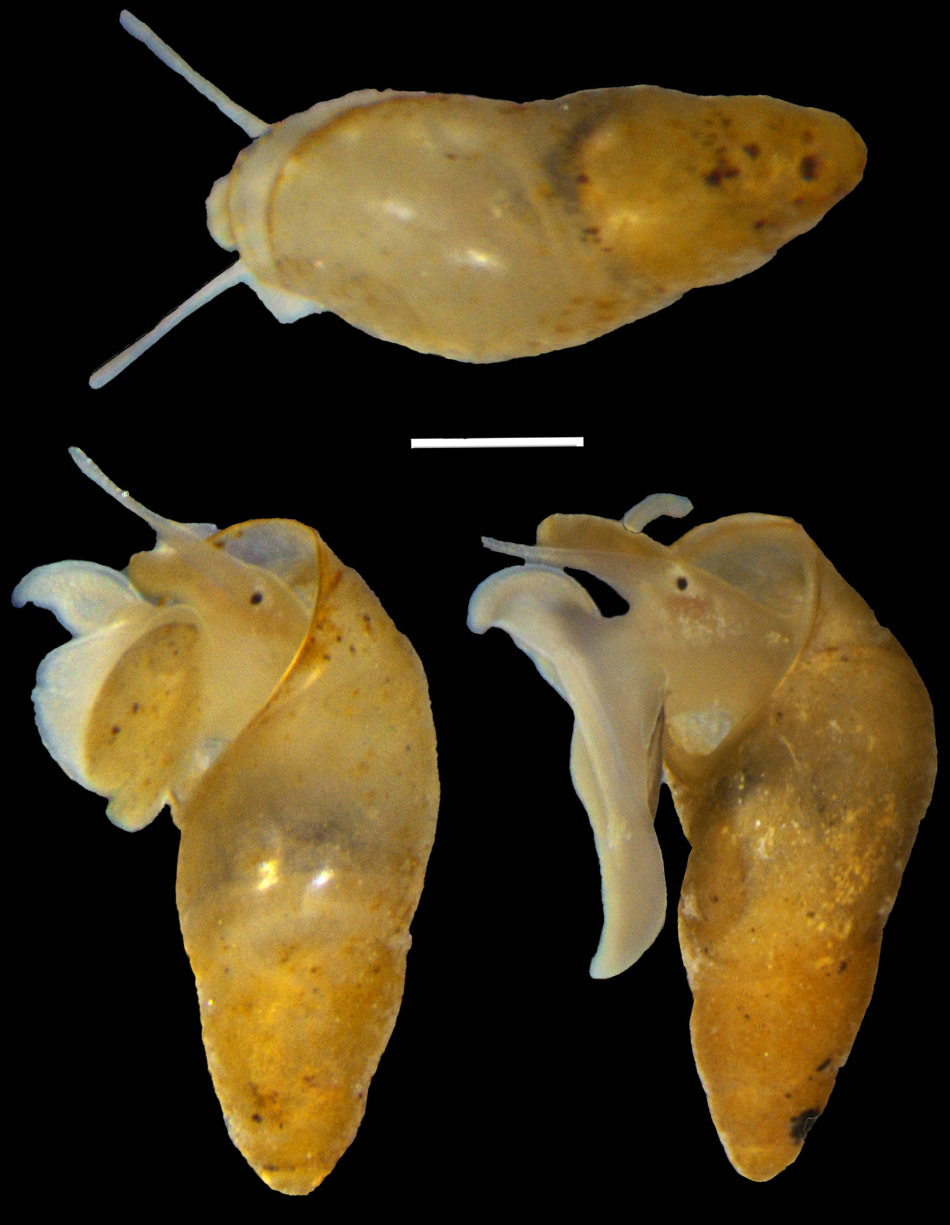
Living *Aenigmula sinensis* sp. nov. Scale bars: 1000 μm.

***Head-foot*** (Fig. 6). Cephalic region pale yellow, darker on the head; periocular areas with pale yellow chromatophores. Posterior margin of foot simple, lacking caudal cirri.

###### Distribution and habitat.

*Aenigmula sinensis* inhabits a rocky habitat in the mid-intertidal zone of muddy flats, where the rocks are partially buried in mud, creating narrow gaps between the rock base and the mud surface; individuals are distributed on the underside of rocks, predominantly within concavities and rough crevices.

###### Etymology.

The specific name *sinensis* refers to the country where the new species was discovered. This represents the first record of the genus *Aenigmula* in China.

###### Remarks.

This species is small-sized and was collected from beneath rocks in muddy intertidal zones, where it occurs in dense aggregations. This marks the first record of the genus in China, and it has not been observed in other regions of the country to date. Compared to the type locality of *A. criscionei* (Rapid Creek, Australia), the two species are geographically distant and exhibit markedly different habitat and climatic characteristics: *A. sinensis* sp. nov. was discovered beneath rocks in muddy intertidal zones under a temperate monsoon climate zone, while *A. criscionei* was collected from a mangrove forest in a tropical savanna climate zone.

In morphology, adults of this species can be readily distinguished from *A. criscionei* (detailed differences are shown in Table 4). Although its subadults closely resemble *A. criscionei* in shell shape, this species can be identified by its pale-yellow shell, more prominent whorls, exceptionally thin aperture, and a straight inner lip without any curvature. Molecular data further support this morphological differentiation and confirm their distinct species status.

**Table 4.** Morphological comparison of the three species of *Aenigmula*.

Character and Habitat	* A. criscionei*	* A. sinensis* sp. nov.	* A. * sp.
Location	Northern Territory, Australia	Shangdong, China	Jiangsu, China
Habitat	Mangrove forest	Rocky reef in muddy intertidal zones	Rocky reef in muddy intertidal zones
Size(length, mm)	2.14–2.93	2.57–3.27	7.70
Shell			
Shape	Ovate-conical	Elongated-conical	Fusiform
Color	Glossy, golden	Pale yellow and translucent	Yellowish-white and translucent; apex pale green (possibly environmentally induced)
whorls	5–6	5–6	6–7
Periphery	Straight-sided to slightly convex	Slightly convex to convex	Slightly convex
Suture	Smooth	Distinct	Smooth
Aperture	Teardrop-shaped	D-shaped	Teardrop-shaped
Protoconch	Dome-shaped	Dome-shaped	Dome-shaped
Sculpture	Smooth except for growth lines	Smooth except for growth lines	Smooth except for growth lines
Radula			
Central tooth	3–4+1+3-4/1+1	3+1+3/1+1	3+1+3/1+1
Lateral tooth	4–5+1+5–6	5–6+1+5	8+1+3–5
Inner marginal	~20	~25	~20
Outer marginal	~20	10-15	Unknown
Operculum	Teardrop-shaped, paucispiral, nucleus eccentric	D-shaped, paucispiral, nucleus eccentric	Unknown

##### Aenigmula

Taxon classificationAnimaliaLittorinimorphaTornidae

sp.

75A62DAE-702B-5071-A2C6-53E4043C93F1

Fig. 7

###### Material.

1 specimen; Nantong, Jiangsu, China; 31°51'36"N, 121°52'12"E; coll. Jiaze Tang; 23 July 2024.

###### Measurements (μm).

Shell height: 7699; Shell width: 3170; length of aperture: 3342; width of aperture: 2046.

###### Diagnosis.

Fusiform, glossy, suture distinct; whorls slightly convex, body whorl inflated; surface smooth except for growth lines; aperture teardrop-shaped, without external varix; imperforate; main cusp of central radular tooth markedly prominent, nearly level with the single pair of basal denticles.

###### Description.

**Figure 7.**
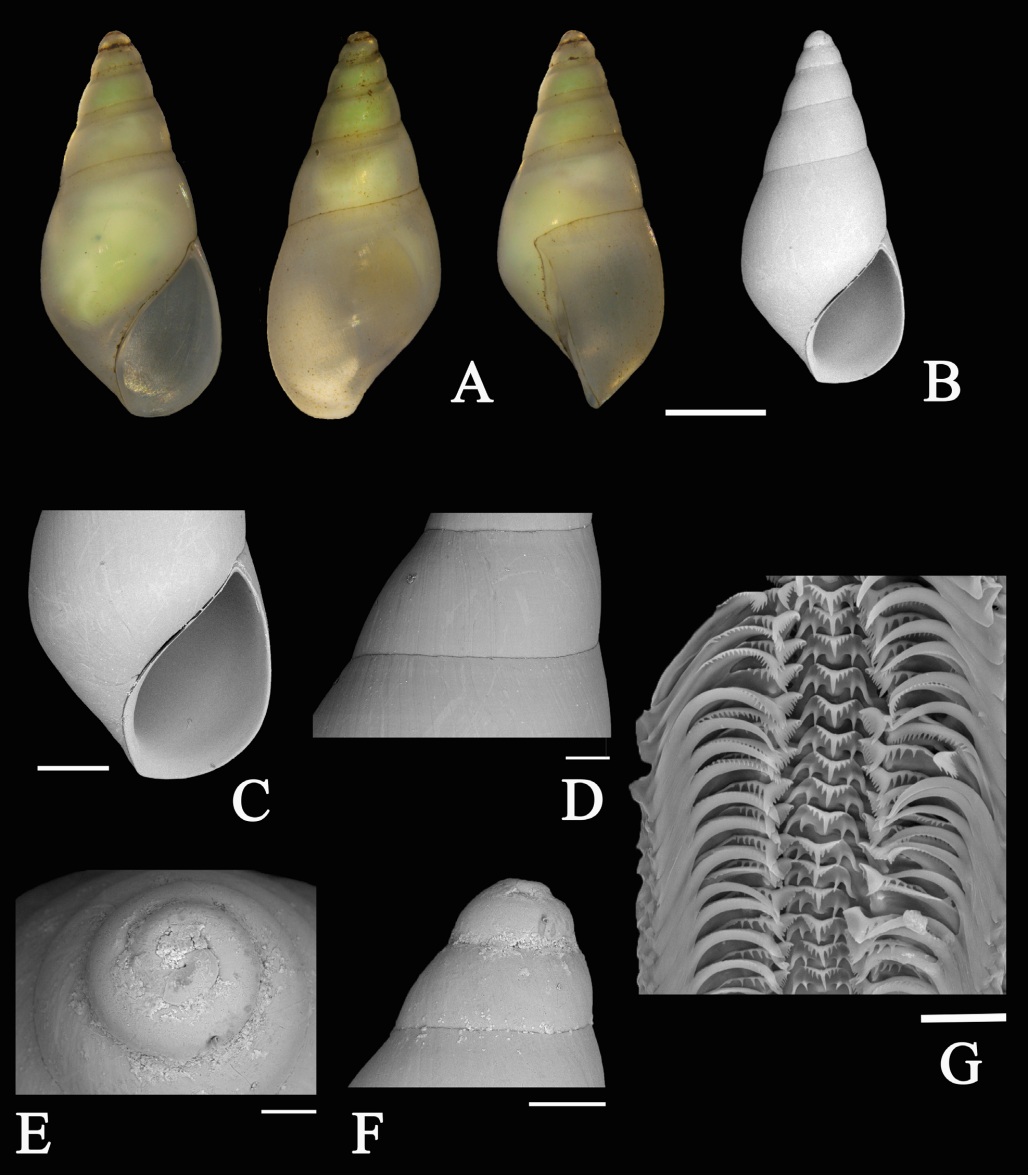
*
Aenigmula* sp. **A**. Three-view photographic documentation of the shell; **B–G**. Scanning electron micrographs: **B**. Apertural view; **C**. Aperture region; **D**. Sculpture; **E**. Protoconch; **F**. Protoconch; **G**. Radula. Scale bars: 1000 μm (**A, B**); 500 μm (**C**); 200 μm (**D, F**); 100 μm (**E**); 20 μm (**G**).

***Shell*** (Fig. 7A–F). Small (~ 8 mm), fusiform, thin but solid, glossy, yellowish-white, and translucent; apex pale green (possibly environmentally induced); suture smooth. Whorls slightly convex, 6–7 in number; body whorl inflated, occupying about 3/5 of shell height. Surface smooth, devoid of sculpture except for growth lines. Aperture teardrop-shaped; outer lip thin; inner lip narrow, smooth, lacking external varix. Imperforate.

***Radula*** (Fig. 7G). Central tooth 3+1+3 / 1 1, trapezoidal, bearing a single slender conical main cusp, flanked on each side by three sharp denticles, with one pair of well-developed, elongated conical basal denticles. Lateral teeth 8+1+3–5, distinctly asymmetrical; inner denticles comb-like and subequal in size; outer denticles sparse and crescentic-conical. Marginal teeth elongated and curved, comprising inner and outer marginal teeth: inner marginal teeth broad-based, bearing ~ 20 denticles externally and fewer internally; outer marginal teeth with denticles confined to the inner side.

***Operculum***. Unknown.

***Head-foot***. Unknown.

###### Distribution and habitat.

*Aenigmula* sp. inhabits a rocky habitat in the mid-intertidal zone of muddy flats, where the rocks are partially buried in mud. Individual specimens attach to concavities on the underside of rocks.

###### Remarks.

This species was discovered incidentally during sampling. Only a single specimen was obtained, which was entirely consumed for DNA extraction. Its collection habitat resembles that of *A. sinensis* sp. nov., but no conspecific individuals were found in the vicinity. Its morphology and radula exhibit distinct characteristics of *Aenigmula*, but it can be readily distinguished from other congeners based on individual size and shell shape. Further detailed differences are presented in Table 4. Furthermore, the independence of this individual is supported based on COI. However, due to the limited specimen availability, it is not possible to obtain duplicate molecular data or specific morphological details of the operculum and soft body. Therefore, this specimen is provisionally identified as *A.* sp. Future sampling efforts will be conducted in relevant regions to supplement missing data and further investigate whether this unknown species represents a potential new species.

## Discussion

### Phylogenetic analysis of the superfamily Truncatelloidea based on mitochondrial genomes

The phylogenetic framework for Truncatelloidea is first presented based on mitochondrial genomes, resolving the superfamily into two major clades (Fig. 2I, II), which is consistent with previous studies based on short genetic segments (Ponder et al. 2014; Takano and Kano 2014; Layton et al. 2019; Goto et al. 2021; Zhang et al. 2024). However, this result is challenged by other studies, mainly regarding the phylogenetic position of *Nodulus contortus* (Golding 2014a, 2014b). In previous studies, *N. contortus* was shown to be separated from other genera within Anabathridae, existing independently from the two major clades. Since the species was not included in the present study, further validation is required to determine whether the anomalous phylogenetic position of *N. contortus* stems from data bias or reflects its taxonomic distinctiveness by increasing the sampling of Anabathridae and obtaining additional sequence data for this species.

Phylogenetic relationships among families within Truncatelloidea were inconsistent across analyses based on different short genetic segments and were also discordant with the topology reconstructed from mitochondrial genomes. Within Clade I, although multiple studies support a closer phylogenetic relationship between Assimineidae and Pomatiopsidae (Golding 2014a, 2014b; Layton et al. 2019; Goto et al. 2021; Zhang et al. 2024), our results indicate that Assimineidae is a sister group to Stenothyridae, which together clusters with Pomatiopsidae. This topological discrepancy likely stems from differences in taxon sampling, as well as the use of mitogenomic data, which provides more phylogenetic information than short gene fragments such as COI, 16S rRNA, and 28S rRNA. Furthermore, Baicaliidae and Bithyniidae are clearly more distantly related to other families, including Tateidae, in contrast to previous studies which positioned Tateidae in a more basal position (Layton et al. 2019; Goto et al. 2021; Zhang et al. 2024). In Clade II, while previous studies recovered a phylogeny in which the clade containing *Aenigmula* is a successive sister group to Clenchiellidae, then to Caecidae, and finally to Tornidae (Ponder et al. 2014; Golding 2014b), our results support the phylogenetic relationship ((*Aenigmula* + Clenchiellidae) + Tornidae) + Caecidae, indicating a clear distinction. Moreover, in previous analyses lacking molecular data for *Aenigmula*, the topology among Clenchiellidae, Caecidae, and Tornidae was unstable. Existing studies have alternately supported Clenchiellidae and Caecidae (Criscione and Ponder 2013), Clenchiellidae and Tornidae (Golding 2014a; Layton et al. 2019; Goto et al. 2021), or Caecidae and Tornidae (Zhang et al. 2024) as sister groups.

Additionally, in our analyses, the monophyly of all families and genera was strongly supported, except for *Assiminea* (Assimineidae). Previous studies have demonstrated that Iravadiidae is polyphyletic (Criscione and Ponder 2013; Golding 2014b; Goto et al. 2021). Although our results strongly support the monophyly of Iravadiidae, the limited sampling from coastal China and the absence of key taxa (such as *Nozeba topaziaca* and *Auricorona queenslandica*) in our molecular dataset necessitate broader sampling to firmly establish the family’s monophyly and phylogenetic position.

### Taxonomic classification of *Aenigmula*

Golding (2014b) was unable to effectively classify *A. criscionei* at the family level and instead established a new genus with this species as the type species, due to insufficient morphological data and the lack of molecular information for most relevant family-level taxa. Our results significantly enhance the understanding of the genus by providing comprehensive morphological and molecular evidence from a new species (*A. sinensis* sp. nov.) and an additional undescribed taxon (*A.* sp.). Although this genus shows simple morphological characteristics, detailed morphological observations still enable effective distinction from groups within Truncatelloidea and Rissooidea that share similar shell shapes and possess smooth surfaces or only a single type of sculpture. Diagnostic features such as shell coloration (*Assiminea sinensis* G. Nevill, 1880 is brownish; *Heleobia isabelleana* (A. d’Orbigny, 1841) is white and translucent); protoconch characters (*Laevicaspia cincta* (Abich, 1859) bears faint granular protrusions); subtle shell surface carving (*Nozeba topaziaca* (Hedley, 1908) and *Schwartziella inscripta* Rolán & Luque, 2000 both exhibit fine spiral grooves on the first whorl); and aperture morphology (*Pisinna compressa* (Laseron, 1956) is oval-shaped; *Botryphallus ovummuscae* (Gofas, 1990) has a thickened aperture) provide crucial evidence for establishing *Aenigmula*’s independent status.

Moreover, molecular phylogenetic evidence also provides a key basis for supporting its distinctiveness. The phylogeny based on concatenated sequences of COI, 16S, and 28S rRNA genes showed that *Aenigmula* is monophyletic with the highest support (PP = 1; BS = 100%). The mitogenomic phylogeny also confirmed the distinct systematic position of *A. sinensis* sp. nov., clearly separating it from all other families of Truncatelloidea.

Based on strong molecular evidence and distinct morphological differentiation of this genus from phylogenetically related families, our study concludes that the taxonomic position of *Aenigmula* holds significant research importance. The genus currently comprises two species: *A. criscionei* and *A. sinensis* sp. nov. Although the limited individual specimens of *A.* sp. preclude its formal description, its existence indicates previously unrecognized diversity within the genus, warranting further investigation. The morphological characteristics of this genus include a glossy and completely unsculptured shell, a dome-shaped protoconch, a simple teardrop-shaped aperture, and highly consistent radular and opercular structures.

A family for the genus *Aenigmula* is not formally proposed in our study, primarily based on the following reasons: (1) The taxonomic utility of genital morphology has been highlighted (Hershler and Ponder 1998); however, due to the lack of anatomical data provided for *A. criscionei* and the limited number of specimens available for *A. sinensis* sp. nov., detailed anatomical characteristics of this genus cannot currently be obtained. Given the simple shell morphology and absence of surface sculpture in this taxon, anatomical analysis is expected to substantially enhance the resolution of taxonomic classification. (2) The limited availability of short genetic segments for the new species and related taxa currently precludes a reliable estimation of its divergence time, thereby failing to provide a robust chronological framework for its phylogenetic position. Therefore, future studies should expand sample collection and further integrate morphological and molecular phylogenetic information to provide compelling evidence for the valid establishment of this family.

## Supplementary Material

XML Treatment for Aenigmula

XML Treatment for Aenigmula sinensis

XML Treatment for Aenigmula

## Additional information

### Artificial Intelligence (AI) use

The authors accept full responsibility for the content of the manuscript, including the disclosure of any use of AI.

No AI tools were used in the preparation of this manuscript.

### Author ORCIDs

J. Tang https://orcid.org/0009-0006-7148-8340

J. Gong https://orcid.org/0009-0000-0083-5495

X. Han https://orcid.org/0009-0002-6607-3002

Y. Fan https://orcid.org/0009-0001-9709-7130

X. Zhang https://orcid.org/0009-0007-6044-6804

L. Kong https://orcid.org/0000-0001-5263-1697

### Data availability

All of the data that support the findings of this study are available in the main text or Supplementary Information.

## Supplementary materials

Supplementary material 1 Supplementary material 1: table SS1, S2 Data type xls Explanation note Short-segmented and mitochondrial data used in the study. https://binary.pensoft.net/file/1650883This dataset is made available under the Open Database License (http://opendatacommons.org/licenses/odbl/1.0/). The Open Database License (ODbL) is a license agreement intended to allow users to freely share, modify, and use this Dataset while maintaining this same freedom for others, provided that the original source and author(s) are credited. Jiaze Tang, Jihang Gong, Xiao Han, Yan Fan, Xinni Zhang, Lingfeng Kong

Supplementary material 2 Supplementary material 2: figures S1–S6 Data type doc Explanation note ABGD analyses, ASAP analyses and bPTP analyses. https://binary.pensoft.net/file/1650884This dataset is made available under the Open Database License (http://opendatacommons.org/licenses/odbl/1.0/). The Open Database License (ODbL) is a license agreement intended to allow users to freely share, modify, and use this Dataset while maintaining this same freedom for others, provided that the original source and author(s) are credited. Jiaze Tang, Jihang Gong, Xiao Han, Yan Fan, Xinni Zhang, Lingfeng Kong

Supplementary material 3 Supplementary material 3: table SS3 Data type xls Explanation note Details of the sampling sites. https://binary.pensoft.net/file/1650885This dataset is made available under the Open Database License (http://opendatacommons.org/licenses/odbl/1.0/). The Open Database License (ODbL) is a license agreement intended to allow users to freely share, modify, and use this Dataset while maintaining this same freedom for others, provided that the original source and author(s) are credited. Jiaze Tang, Jihang Gong, Xiao Han, Yan Fan, Xinni Zhang and Lingfeng Kong
