# Supplementary figures and images for: Mitogenomic phylogeny of Truncatelloidea with description of Aenigmula sinensis Tang, Han & Kong, sp. nov. (Mollusca, Gastropoda, Littorinimorpha, Truncatelloidea)
Source: Zookeys. 2026 May 19;1279:357–78. doi: 10.3897/zookeys.1279.183841 (PMC13213388; doi:10.3897/zookeys.1279.183841)

**ABGD analyses:**


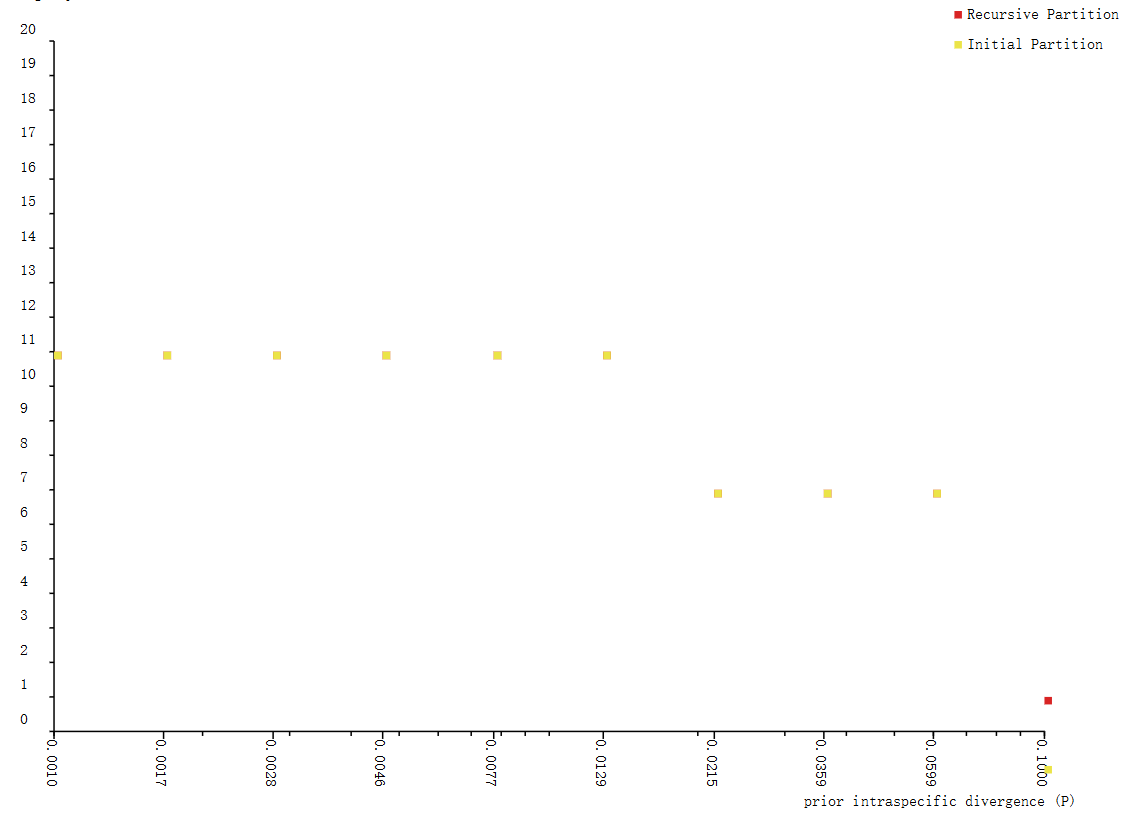

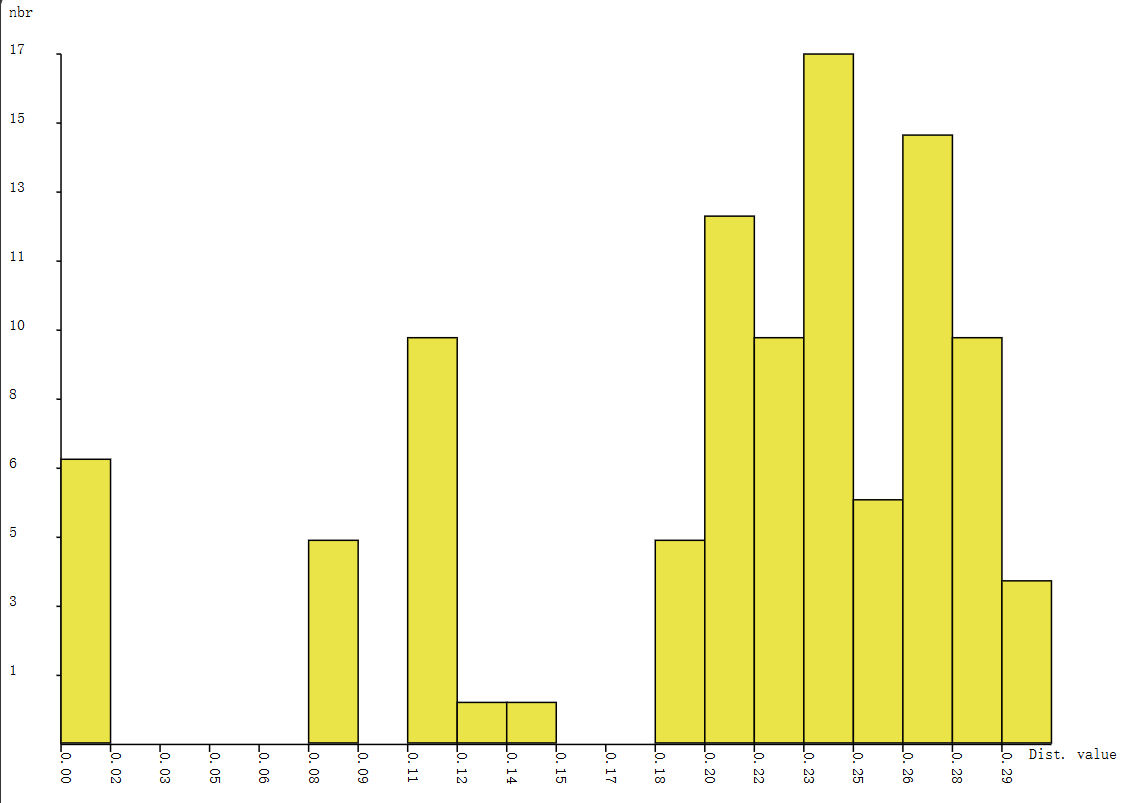


**ASAP analyses:**


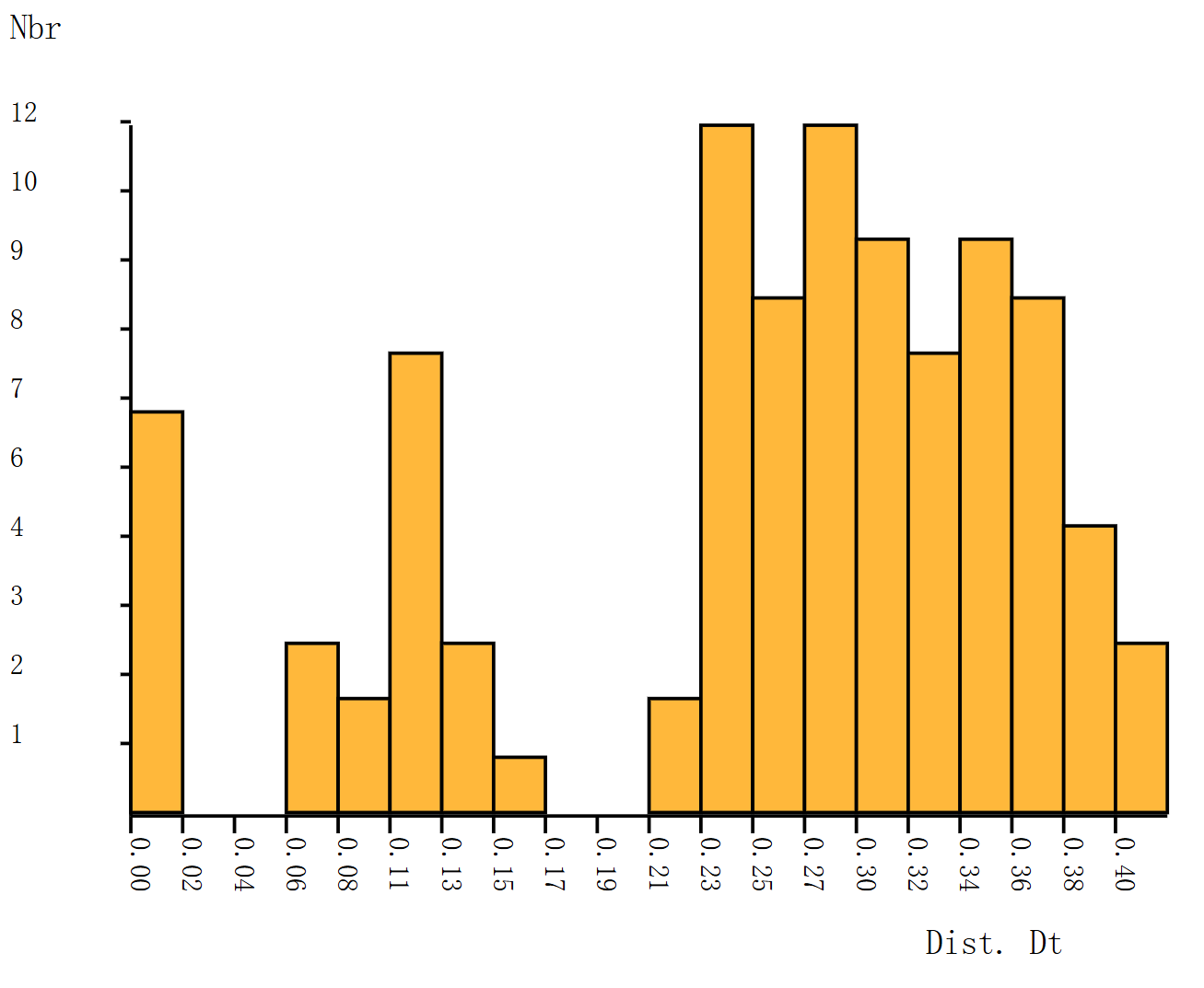


**
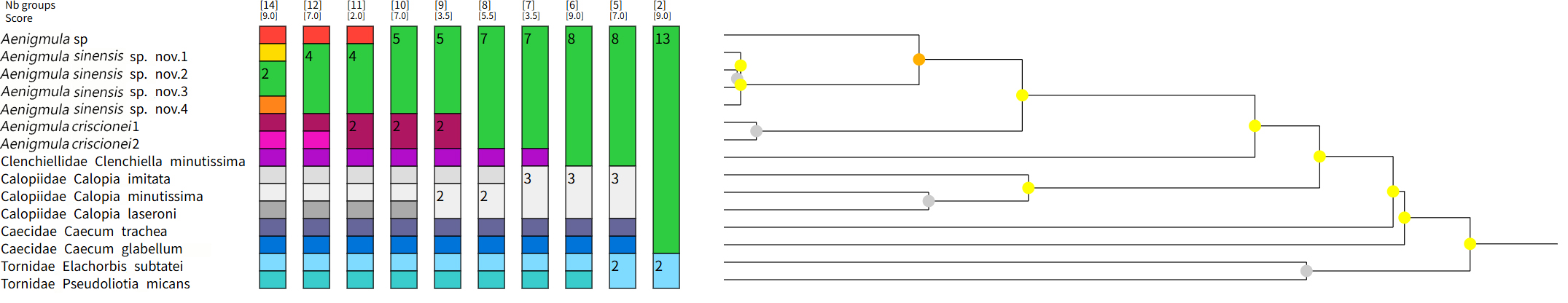
**

**bPTP analyses:**


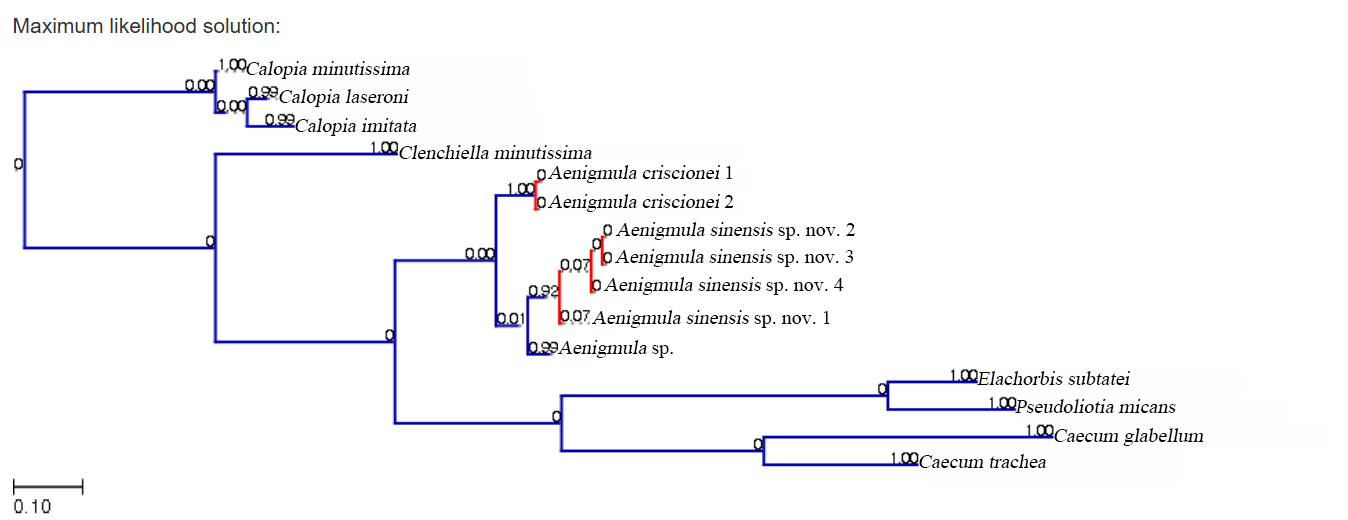


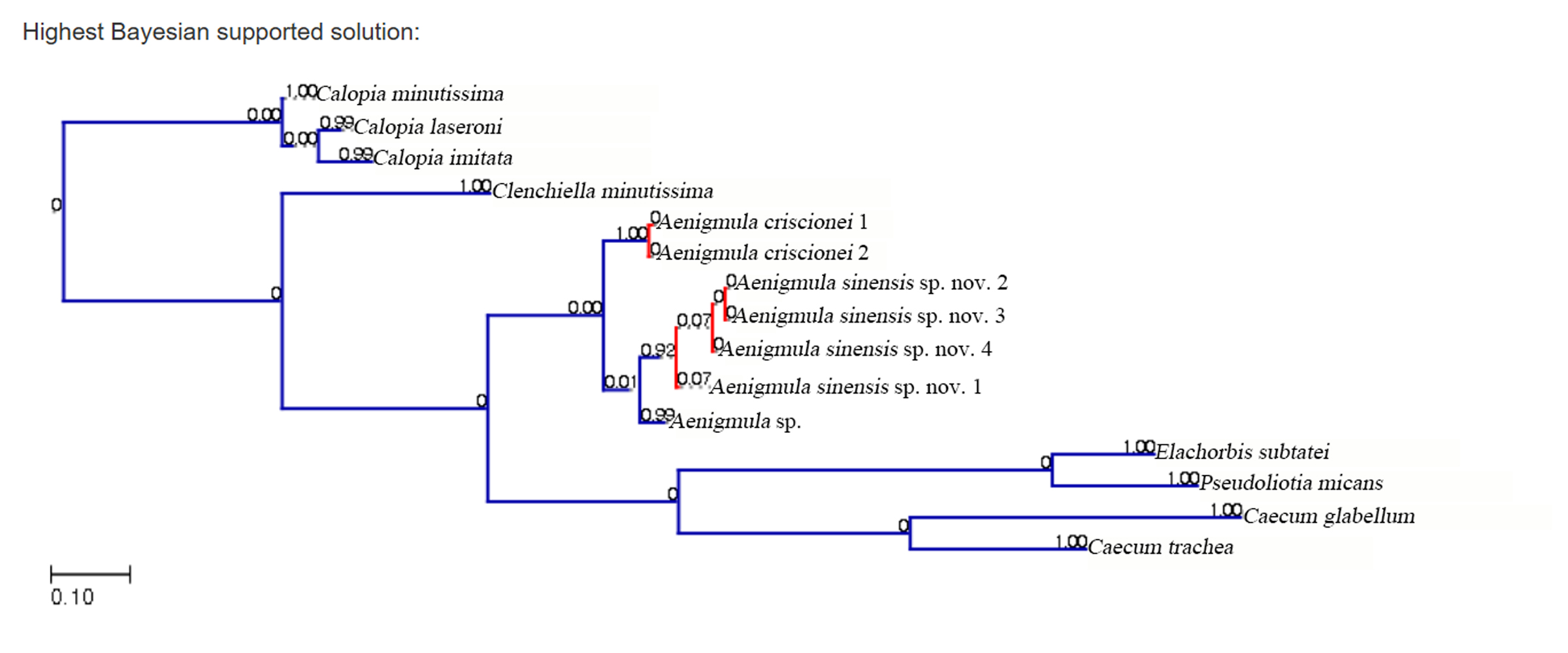

Supplement: Supplementary material 2 — figures S1–S6 [file zookeys-1279-357_article-183841__-s002.doc]
